# Ceria Nanoparticles Mitigate the Iron Oxidative Toxicity of Human Retinal Pigment Epithelium

**DOI:** 10.7759/cureus.9675

**Published:** 2020-08-11

**Authors:** Jason Kieffer, Sushant Singh, Baltej S Dhillon, Udit Kumar, Saad Shaikh, Son Ho, Sudipta Seal

**Affiliations:** 1 Ophthalmology, University of Central Florida College of Medicine, Orlando, USA; 2 Advanced Materials Processing and Analysis Center, Nanoscience Technology Center, Dept. of Materials Science and Engineering, University of Central Florida, Orlando, USA; 3 Ophthalmology, Orlando VA Medical Center, Orlando, USA; 4 Materials Science Engineering, Advanced Materials Processing and Analysis Center, Nanoscience Technology Center, Dept. of Materials Science and Engineering, University of Central Florida, Orlando, USA

**Keywords:** ceria, oxidation, reactive oxygen species, retina, macular degeneration, iron, amd, nanoparticle

## Abstract

Oxidative injury is implicated in retinal damage observed in age-related macular degeneration (AMD), as well as other degenerative conditions. Abnormally elevated levels of iron accumulation within the retinal pigment epithelium have been detected in eyes with AMD, and it is suspected to play a role in the pathogenesis through the production of reactive oxygen species (ROS). Ceria nanoparticles (CNP) have the ability to scavenge ROS. This study sought to evaluate the ability of CNP to mitigate iron-induced oxidative stress and assess cell viability in the human ARPE-19 cell line in vitro. Cell viability was measured by an MTT (3-(4,5-Dimethylthiazol-2-yl)-2,5-diphenyltetrazolium bromide) assay and compared between experimental groups undergoing 48-hr exposure to a ferrous iron solution with and without 24-hr CNP pre-treatment. The CNP effect on ROS formation was evaluated additionally by H2DCFDA (2,7-dichlorodihydrofluorescein diacetate) fluorescent probe assay and superoxide dismutase assay. CNP demonstrated a three-fold increase in cell viability and a reduction in ROS generation. The results show a promising treatment modality for diseases causing oxidative damage in the eye.

## Introduction

Retinal diseases, such as age-related macular degeneration (AMD), lead to vision loss and, eventually blindness as a result of chronic degenerative changes. The pathogenesis of AMD is multifactorial; however, growing evidence suggests a contributing role of oxidative stress in the destruction of photoreceptor cells [1-2]. Significantly greater amounts of iron deposition within retinal pigment epithelial cells (RPE) and Bruch’s membrane have been demonstrated in donor retinas with AMD when compared to age-matched healthy donor retinas [3-4]. The potential for iron to generate reactive oxygen species (ROS) proposes a mechanism in which the excess deposition found in AMD retinas is relevant to the progression of the disease [5]. Local inflammation can also stimulate polymorphonuclear leukocytes and macrophages, which demonstrate the ability to release iron from carrier proteins, such as ferritin, through their induction of superoxide radicals [6].

In addition to the damage caused by chronic deposition, iron and other reactive metals are a common source of acute intraocular foreign bodies. Iron particulates retained within the globe from trauma can lead to developing the condition siderosis bulbi, posing significant risks to a patient’s health and vision [7]. Barriers to prompt surgical removal can occur, including rural settings with limited expertise, military personnel in combat environments, and cases determined to be inoperable. This places these individuals at higher risk of eye-related complications and loss of vision.

Ceria nanoparticles (CNP) have been used in various commercial and industrial functions. There has been expanding research of CNP in the medical field as a therapeutic remedy for oxidative stress disorders. For example, there have been investigations into their potential benefit in conditions like type II diabetes mellitus, neuronal injury, and iatrogenic injury of the gastric epithelium from radiation [8-11]. CNP consist of a mixture of the metal cerium (Ce) in the 3+ and 4+ oxidation state present on the surface of the nanoparticle. These CNP possess regenerative catalytic capabilities for scavenging ROS [9]. Synthesized preparations of CNP have demonstrated catalytic activity similar to both superoxide dismutase (SOD) and catalase [12-13]. SOD acts to convert superoxide radicals O_2_•- into H_2_O_2_ and O_2_. Meanwhile, catalase reduces hydrogen peroxide to H_2_O and O_2_ [12-13]. The nanoparticle size facilitates these CNP to easily pass through cellular and mitochondrial membranes where ROS can be present. Animal models have suggested the safe administration of CNP to retinal tissue, without damage to structure or function, by intraocular injection [14-15]. This safety has also been demonstrated when applying CNP in vitro to human lens epithelial cells [16].

We designed an in vitro microplate model to test the ability of CNP to protect human cell line RPE cells, ARPE-19, exposed to an environment of oxidative stress caused by a ferrous iron solution. The ability of CNP to prevent ROS generation and SOD enzymatic activity was further studied to determine the magnitude of the effect. The results of this study further contribute to the growing evidence surrounding CNP as a potentially valuable therapeutic agent, in particular, for retinal diseases linked to oxidative injury.

## Materials and methods

Ceria nanoparticles synthesis and characterization

CNP were synthesized in 5mM concentration and size range of 3-5 nm using the previously reported wet chemistry protocol [17]. The size of the CNP utilized was selected following the results of this prior investigation, which demonstrated that the ultra-small 3-5-nm sized CNP were more efficiently taken up by cellular membrane mechanisms in comparison to larger CNP [17]. In brief, the Ceria precursor salt (99.999% purity grade level) was mixed in ultrapure water and hydrogen peroxide was added to induce the oxidation of cerium ions into ultrasmall cerium nanomaterial. This prepared CNP was further characterized for size, surface charge, zeta potential, and biochemical enzyme mimetic SOD activity using a zeta sizer (Nano-ZS from Malvern Instruments, Malvern, United Kingdom) and the SOD assay kit (Sigma Aldrich, Kit #19160-1KTF, St. Louis, Missouri). A high-resolution transmission electron microscope (HR-TEM, Philips Tecnai, Philips Healthcare, Andover, Massachusetts) was used to accurately measure the CNP size.

ARPE-19 cells culture and maintenance

The human RPE cell line, ARPE-19, were acquired from the American Type Culture Collection (Cell line Catalogue no. ATCC-CRL-2302). Cells were maintained in Dulbecco’s Modified Eagles Medium: Ham’s F12 Nutrient Mix (ATCC, 30-2006) with 10% fetal bovine serum (ATCC, 30-2021) and 1% antibiotic/antimycotic solution (Thermo Fisher Scientific, 15240096, Waltham, Massachusetts). Cells were maintained in an incubator at 37^o^C and 5% CO_2_.

Evaluating iron and ceria nanoparticle cytotoxicity in the ARPE-19 cell line

The ferrous iron solution was prepared by dissolving FeSO_4_·7H_2_O (Sigma Aldrich, F8633) in serum-free media. The concentration of the ferrous iron solution used to assess cell rescue, 7.5 mM, was selected following preliminary toxicity trials of ARPE-19 cells exposed to a range of concentrations. Lower concentrations did not reliably produce sufficient cell death necessary to evaluate the benefit of CNP treatment. Concentrations in excess of 10 mM meanwhile resulted in insoluble precipitants remaining in solution. Preparing the iron solution at a concentration of 7.5 mM balanced these two concerns and allowed for the effective evaluation of the CNP ability to improve cell viability. ARPE-19 cells were exposed to the ferrous iron solution for 48 hr. Cell viability was measured in four replicates by MTT (3-(4,5-Dimethylthiazol-2-yl)-2,5-diphenyltetrazolium bromide) assay after 48 hr. CNP pre-treatment was performed following a protocol designed by a previous study [11]. ARPE-19 cells were exposed to a CNP solution of 200 µM in serum-free media for 24 hr. The concentration of the CNP solution was selected as a moderate dose for focused study following preliminary testing of low (10 µM) to high (500 µM) dose concentrations. The moderate dose concentration (200 µM) optimized antioxidative effect and avoided potential interference of cellular mechanisms. The CNP solution was completely removed following treatment, cells were washed with phosphate-buffered saline, and then exposed to the ferrous iron solution for 48 hr. The potential cytotoxicity from CNP was also assessed by a group exposed to CNP solution for 48 hr in the absence of an iron solution. Absorbance measurements were obtained by a microplate reader. A comparison of experimental conditions to control was performed to determine the relative increase or decrease in cell viability.

ROS production

H2DCFDA (2,7-dichlorodihydrofluorescein diacetate) assay (Thermo Fisher, D399) was utilized to assess ROS production in experimental groups and control across four replicates. The protocol was utilized as cited in the literature [18]. H2DCFDA stock concentration prepared with dimethyl sulfoxide was diluted in media to a concentration of 1 µM and incubated in the dark for 30 minutes. Measurement of fluorescence was obtained by a microplate reader using excitation at 485 nm and emission at 520 nm. ARPE-19 cells were seeded at a density of 30,000 cells per well in a fluorescence microplate for this analysis.

Statistical analysis

Data analysis was performed using Excel software, version 16.0 (Microsoft Corporation, Redmond, Washington). Statistical significance was determined by a p-value of <0.05. Figures were prepared using Origin software (OriginLab Corporation, Northampton, Massachusetts) unless otherwise specified.

## Results

**Figure 1:**
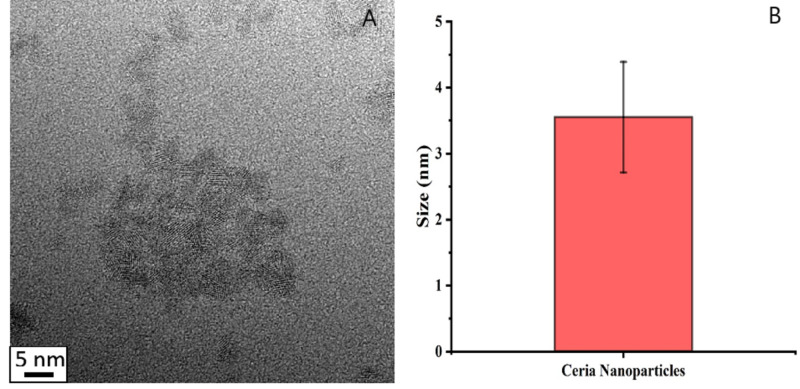
CNP synthesis A: HR-TEM image of CNP synthesis using the wet chemistry approach; B: Average size of the CNP represented by histogram analysis. CNP: ceria nanoparticles

The CNP were synthesized in the size range of 3-5 nm, as analyzed from the HR-TEM image. Figure 1 presents the HR-TEM image of CNP synthesized using wet chemistry and histogram analysis of the size of the nanoparticles prepared using ImageJ software (Bethesda, Maryland). This indicates CNP creation following the highly optimized protocol for CNP synthesis, which has been reported numerous times previously [19-20].

**Figure 2:**
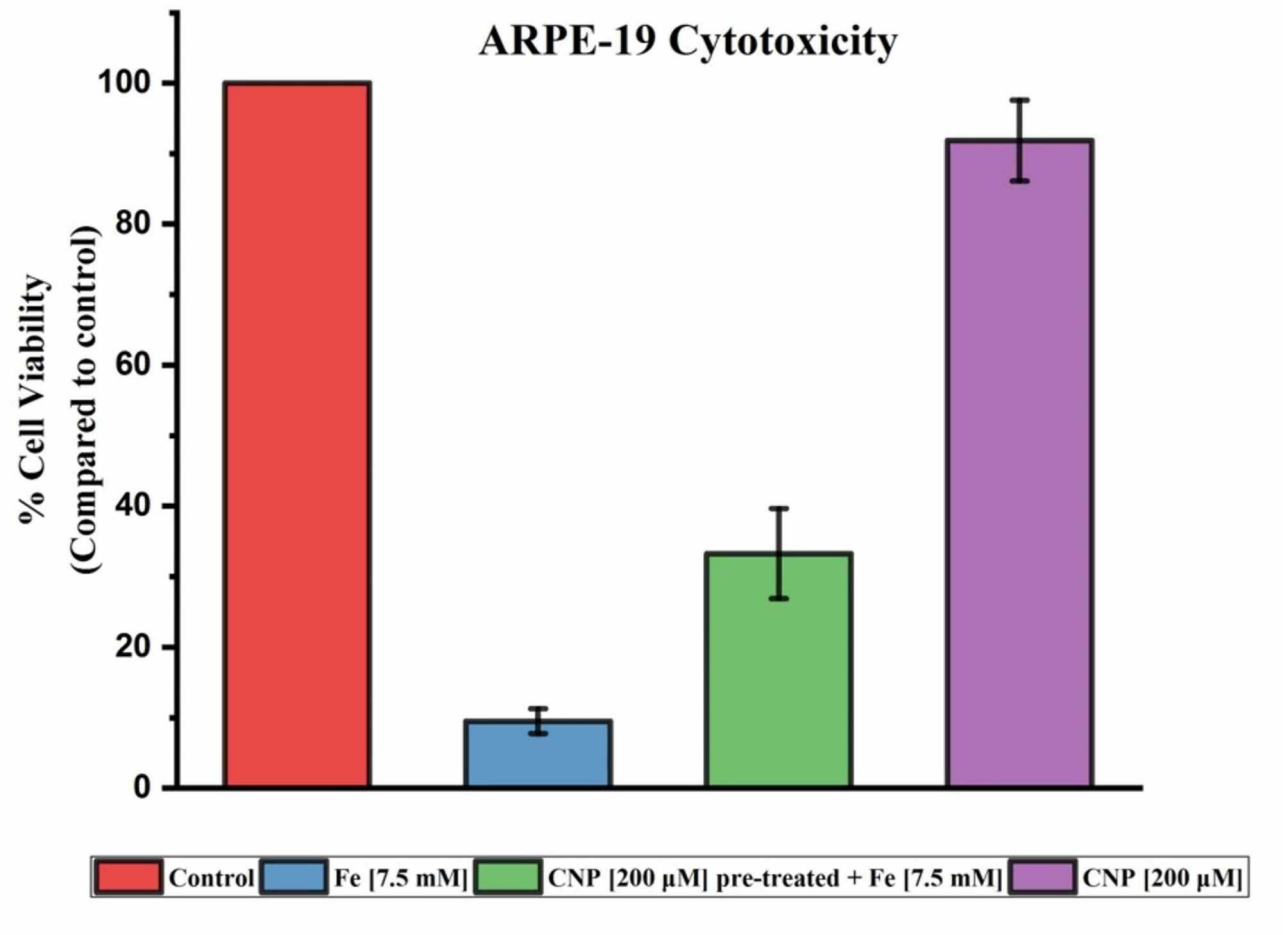
ARPE-19 cytotoxicity MTT assay of cytotoxicity after 48 hr exposure to Fe solution (7.5mM), Fe solution (7.5mM_ following 24 hr pre-treatment of CNP (200 µM), and 48 hr exposure of CNP (200 µM). The data presented here are the mean value of replicate experiments performed with standard deviation. CNP: ceria nanoparticles; MTT: 3-(4,5-Dimethylthiazol-2-yl)-2,5-diphenyltetrazolium bromide

The graph in Figure 2 demonstrates the results of the MTT assay evaluating cytotoxicity after 48 hr. The mean viability of the replicates exposed to Fe (7.5 mM), Fe (7.5 mM) following CNP pre-treatment, and CNP (200 µM) was 9.5% ± 1.8%, 33.3% ± 6.4%, and 91.9% ± 5.7% with respect to control, which is considered 100% viable and is without any treatment. Differences in viable cell populations between iron-exposed groups with CNP pre-treatment and without were compared by a single-tailed t-test and this resulted in a statistically significant difference, with p=0.0047. 

**Figure 3:**
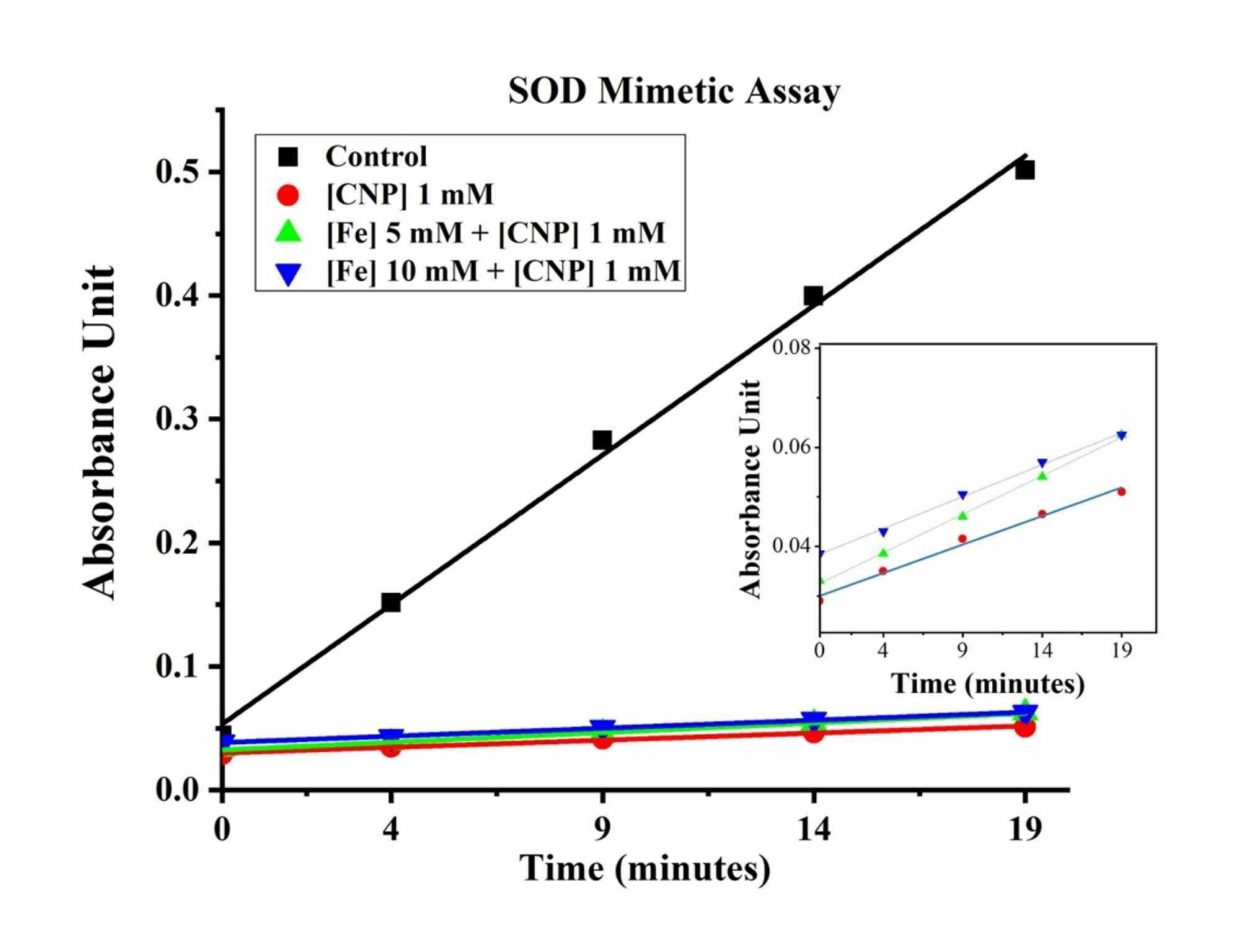
SOD Mimetic Assay SOD assay evaluating the generation of superoxide radicals in the presence of CNP (1 mM), CNP (1 mM) and Fe solution (5 mM), CNP (1 mM) and Fe solution (10 mM), and control. The data presented here are the absorbance measured each minute for 20 cycles (including baseline measurement). SOD: superoxide dismutase; CNP: ceria nanoparticles

To estimate the antioxidative potential for the synthesized CNP, SOD mimetic activity was evaluated using the CNP in combination with an iron solution. The results of the SOD assay are represented in Figure 3. As shown, the CNP remain unaffected by the presence of iron in the surrounding medium. The control group experienced a linear increase in absorbance, with measurements of 0.045 at 0 min and 0.494 at 19 min, or a range of 0.449. Experimental groups had only a slight increase from baseline with a range of less than 0.03.

**Figure 4:**
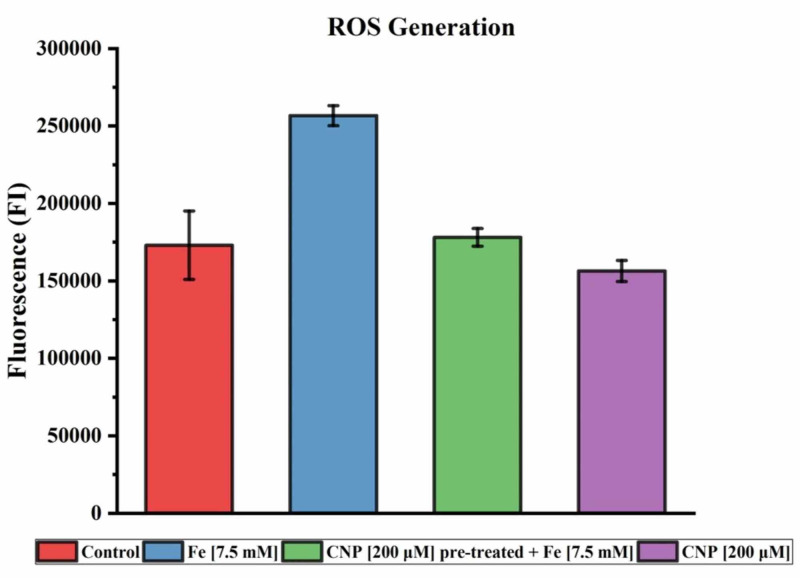
ROS generation H2DCFDA assay of ROS production after 30 min exposure to Fe solution (7.5 mM), Fe solution (7.5 mM) following 24 hr pre-treatment exposure of CNP (200 µM), and CNP (200 µM). The data presented here are the mean value of replicate experiments performed with standard deviation. CNP: ceria nanoparticles; ROS: reactive oxygen species

The results of the H2DCFDA assay evaluating the ROS generation in the ARPE-19 cell line are reported in Figure 4. Mean fluorescence intensity (FI) of the replicates exposed to Fe (7.5 mM), Fe (7.5 mM) following CNP pre-treatment, and CNP (200 µM) were 256,708 ± 6,481, 178,117 ± 5,741, and 156,417 ± 6,850, respectively. The control mean FI was 173,026 ± 22,110. A single-tailed t-test comparing iron-exposed groups with and without pre-treatment demonstrated a statistically significant difference with p=7.9x10^-5^. No statistically significant difference was detected between the control and CNP-treated groups.

## Discussion

In our study, CNP were effectively synthesized in the size range of 3-5 nm (Figure 1). The CNP did not exhibit a toxic effect on ARPE-19 cells during our study period (Figure 2). There was a small deviation of absorbance amounting to 8% on average reduction of a cell population when compared to control. This reduction was minor and can be accounted for by experimental error in the quantity of cells seeded or an effect of the acidic pH of the CNP solution. CNP pre-treatment of the ARPE-19 cells resulted in an increase in cell viability that reached statistical significance (p=0.0047). This demonstrates that CNP can improve ARPE-19 cell survival, as the cell population was, on average, over three times greater. It indicates the CNP's ability to pass through cellular membranes to function as a ROS scavenger within ARPE-19, as the solution had been removed and cells washed before being exposed to the iron medium. The ability of smaller CNP (3-5 nm) to permeate the cell membrane without much energy consumption by uptake pathways suggests a potential for use in nanotherapeutics [17].

The increasing absorbance in control during the SOD assay signifies superoxide radical formation causing a reaction with the chromogen. In comparison, there was minimal inflection from baseline absorbance in the CNP test groups (Figure 3). This indicates that the SOD mimetic activity of the nanoparticles prevented superoxide radical creation and that CNP enzymatic activity was not disrupted by the presence of iron.

When evaluating ROS production by H2DCFDA assay, the presence of iron contributed to a sizeable amount of oxidation, as anticipated (Figure 4). Oxidation was increased by a factor of 1.5 as compared to control. Utilizing the CNP pre-treatment effectively mitigated this ROS production, essentially in its entirety. A comparison of these two groups demonstrated a statistically significant difference in ROS generation (p= 7.9x10-5). The results of the SOD assay and the H2DCFDA assay demonstrate the nanoparticle's powerful ability to reduce oxidative species.

As noted above, CNP did not exhibit any cytotoxic effects on the ARPE-19 cells. The safety profile observed with our study aligns with prior studies demonstrating the safe application of CNP to various cell lines [8,10-11,14-16]. Furthermore, CNP are retained within the retina without evidence of inflammation or destruction to tissue for up to a year in duration [14-15]. The theoretical role for CNP to mitigate oxidative stress within the retinal tissue of patients would likely be through intravitreal injection or potentially a CNP-eluding implant. Further investigation into CNP as a therapeutic agent in conditions linked to oxidative damage like AMD and metallic intraocular foreign bodies where limited treatment options exist is warranted. This study is limited by its in vitro model, which is unable to replicate the complex cellular milieu of human RPE cells. Future studies are needed to better understand the antioxidative effects of CNP and further evaluate its safety and efficacy.

## Conclusions

Our study sought to evaluate the potential for CNP to mitigate the iron oxidative toxicity of RPE cells in vitro. ARPE-19 cells exposed to iron and treated with CNP demonstrated improved cell viability and reduced ROS formation. Furthermore, the performance of a SOD assay confirmed one of the enzymatic mechanisms that have been proposed for the CNP’s antioxidant properties. Future studies are warranted to further explore the treatment potential of CNP in oxidative retinal diseases.
